# Excellent Fireproof Characteristics and High Thermal Stability of Rice Husk-Filled Polyurethane with Halogen-Free Flame Retardant

**DOI:** 10.3390/polym11101587

**Published:** 2019-09-28

**Authors:** Huong T.Q. Phan, Binh T. Nguyen, Lam H. Pham, Chi T. Pham, Thi Vi Vi Do, Cuong N. Hoang, Nguyen Ngan Nguyen, Jinhwan Kim, DongQuy Hoang

**Affiliations:** 1 Department of Polymer and Composite Materials, Faculty of Materials Science and Technology, University of Science, Vietnam National University, HoChiMinh 700000, Vietnamntbinhh0707@gmail.com (B.T.N.); ptchi@hcmus.edu.vn (C.T.P.); dtvvi@hcmus.edu.vn (T.V.V.D.); 2 Department of Polymer Chemistry, Faculty of Chemistry, University of Science, Vietnam National University, HoChiMinh 700000, Vietnam; hncuong@hcmus.edu.vn; 3 Department of Chemical Engineering, Pohang University of Science and Technology, Pohang 37673, Korea; ngannguyen@postech.ac.kr; 4 Department of Polymer Science and Engineering, Sungkyunkwan University, Suwon, Gyeonggi 16419, Korea

**Keywords:** flame retardancy, cone calorimetry, polyurethane/rice husk composite foam, thermal stability, moisture absorption

## Abstract

The thermal stabilities, flame retardancies, and physico-mechanical properties of rice husk-reinforced polyurethane (PU–RH) foams with and without flame retardants (FRs) were evaluated. Their flammability performances were studied by UL94, LOI, and cone calorimetry tests. The obtained results combined with FTIR, TGA, SEM, and XPS characterizations were used to evaluate the fire behaviors of the PU–RH samples. The PU–RH samples with a quite low loading (7 wt%) of aluminum diethylphosphinate (OP) and 32 wt% loading of aluminum hydroxide (ATH) had high thermal stabilities, excellent flame retardancies, UL94 V-0 ratings, and LOIs of 22%–23%. PU–RH did not pass the UL94 HB standard test and completely burned to the holder clamp with a low LOI (19%). The cone calorimetry results indicated that the fireproof characteristics of the PU foam composites were considerably improved by the addition of the FRs. The proposed flame retardancy mechanism and cone calorimetry results are consistent. The comprehensive FTIR spectroscopy, TG, SEM, and XPS analyses revealed that the addition of ATH generated white solid particles, which dispersed and covered the residue surface. The pyrolysis products of OP would self-condense or react with other volatiles generated by the decomposition of PU–RH to form stable, continuous, and thick phosphorus/aluminum-rich residual chars inhibiting the transfer of heat and oxygen. The PU–RH samples with and without the FRs exhibited the normal isothermal sorption hysteresis effect at relative humidities higher than 20%. At lower values, during the desorption, this effect was not observed, probably because of the biodegradation of organic components in the RH. The findings of this study not only contribute to the improvement in combustibility of PU–RH composites and reduce the smoke or toxic fume generation, but also solve the problem of RHs, which are abundant waste resources of agriculture materials leading to the waste disposal management problems.

## 1. Introduction

Rigid polyurethane foam (PUf) is a lightweight material, which can be used in various applications. It has been mostly used in building as a thermal insulation material because of its low thermal conductivity, low density, high compressive strength, and high energy absorption capacity [1,2,3,4,5,6,7,8]. To increase the mechanical strength, acoustic attenuation, and load bearing, inorganic fillers are often added to PUf formulations [9]. Natural filler-reinforced PUf composites have been recently studied for applications in the building and automotive industries [10,11,12,13,14,15]. Among the natural fillers, wood flour or wood fibers [16,17,18,19] are intensely investigated and widely employed in industrial applications. However, PUf and PUf composites are highly flammable materials. Upon exposure to an ignition source, they ignite almost instantaneously and burn rapidly, releasing large amounts of heat and smoke, which may lead to burns and suffocation. The reductions in the amount of heat, smoke release, and release rate through flame retardation modification are important for fire safety during PUf usage. Various applications require a high flame retardancy. Considering the low flame resistances, which hinder their practical applications, fire retardants should be added in the above materials [20,21,22,23,24]. Recently, the number of publications on the flame retardancy of PUf has considerably increased. The studies have focused on expandable graphite (EG) as an intumescent flame-retardant (FR) additive for PUf [25,26,27,28]. However, an effective flame retardancy can be achieved by the addition of a high level of EG. In addition, the required processing may be challenging and the high loading of EG may affect the mechanical properties of the materials. Furthermore, the issues related to the large sizes of the EG particles and low compatibility between the EG and polymer matrix should be overcome. Ye et al. [23] studied the fire retardation of rigid PUfs using fine expandable graphite–poly(methyl methacrylate) (pEG–PMMA) composite particles as a fire retardant. They reported that the compressive strength, modulus, and flame retardancy increased upon the 10 wt% loading of pEG-PMMA. Good flame retardation properties of PUfs have also been obtained by combining EG and phosphate or other FRs [23,24,25,26,27,28,29]. In addition, the synergistic effect of graphene oxide and melamine polyphosphate provided a good flame retardancy [30]. The results of cone calorimetry tests showed the reductions in peak heat release rate, total heat release rate, and total smoke production, compared to those obtained using a neat PUf.

The use of a nonhalogen FR in different PUf composite applications is significant because of the large amounts of toxic and corrosive gases and smoke released by not only the PUf but also the halogen-based FR decomposition. Therefore, it is necessary to develop halogen-free additives. Phosphorus-based FRs are the best candidates for polymeric materials. Among the phosphorus-based FRs commercially available on the market, aluminum diethylphosphinate (Exolit OP), having a high char-forming ability, is the most effective nonhalogen FR. It has a high phosphorous (P) content, effective fire retardancy, and thermal stability at a high temperature. This FR operates mainly through gas phase wherein the phosphorus moieties generated during the decomposition convert to radical-capturing species, which then quench the flame. OP also acts in a condensed phase and leaves residual char during the combustion.

Aluminum hydroxide (ATH) has also been used as a cost-efficient nonhalogen FR and filler for polymer materials. The decomposition of ATH is an endothermic single-step reaction, which releases water (that acts as a cooling agent and/or dilutes the surrounding air) and leaves an aluminum oxide solid contributing to the formation of a char layer that acts as a barrier protecting the polymer from the fire.

Currently, the use of renewable resources has attracted large attention for economically appropriate applications to sustain or even improve the quality of life [9]. It is predicted that approximately 10% and 50% of the petrochemical-based materials will be replaced by renewable plant resources by 2020 and 2050, respectively [31,32,33]. Agricultural wastes, such as rice husks (RHs), are valuable raw materials for lignocellulosic-based production [31]. However, to the best of our knowledge, the flame retardancy of the PU–RH composite has not been extensively investigated.

Vietnam is one of the largest rice exporters. In 2018, Vietnam exported more than 6 million tonnes of rice. As more rice was exported, more RH was created. Consequently, extensive studies should be carried out to convert the rich natural resource into useful materials. In this paper, we report the application of RH as a filler for PUf, with a particular focus on the improvement in flame retardancy of PU–RH.

Based on the previous studies summarized above, the addition of a coadditive/char-forming material along with the phosphorus-based FR could provide an effective flame retardancy. In this study, a single phosphorus-based FR compound (OP), having a high phosphorus content and single most used inorganic filler (ATH), were employed to suppress the PU–RH composite flammability. The thermal behaviors of these materials were evaluated by thermogravimetric analysis (TGA), while their surface morphologies were investigated by field-emission scanning electron microscopy (FE-SEM). Cone calorimetry, limiting oxygen index (LOI), and UL-94 burning tests were carried out to determine the flame retardancies of the composites, whereas the residual chars were characterized by Fourier-transform infrared (FTIR) spectroscopy, FE-SEM, and X-ray photoelectron spectroscopy (XPS). The densities, compressions, and moisture sorption isotherms were also measured and analyzed.

## 2. Materials and Methods

### 2.1. Materials

Polyol (Voracor CR 765) was supplied by Dow Chemical, Guangzhou, China (hydroxyl value: 360 mg KOH/g; viscosity and density of 800 mPas and 1.12 g/cm^3^ at 20 °C, respectively). Methylene diphenyl diisocyanate (MDI) was obtained from Dow Chemical (Voracor CE101; 31.0% NCO, viscosity and density of 210 mPas and 1.23 g/cm^3^ at 25 °C, respectively). Raw RHs (0.18–0.35 mm) were purchased in Southern Vietnam. OP (Exolit OP 1240) was supplied by Clariant, Munich, Germany. ATH was purchased from Guangdong Guanghua Sci-Tech Co., Ltd., Guangdong, China. Distilled water was used as blowing agent.

### 2.2. Preparation of PU-RHs with and without FRs

PU–RHs were prepared in a mold with the dimensions 150 × 150 × 50 mm^3^. Polyol, water as a foaming agent, RH, and FR (Table 1) were mixed together for 20 s in a container using a high-speed mechanical stirrer (1000 rpm). MDI was then added and the mixture was then stirred for 15 s. The resultant mixture was immediately poured into a mold and the lid was quickly closed. After 30 min, the foam was removed from the mold and left at room temperature for 24 h. The PU–RH sample without FR loading was also prepared by the same procedure.

### 2.3. Characterization Equipment

#### 2.3.1. Spectroscopic Analysis: 

XPS measurements were performed with a Kratos AXIS HSi spectrometer using a monochromatized Al K_α_ X-ray source (1486.6 eV) at a constant dwell time of 100 ms (energy of 40 eV). The anode voltage and current were set to 15 kV and 10 mA, respectively. FTIR-ATR spectra were recorded using a JASCO FT/IR-6600 instrument (Tokyo, Japan) in the wavenumber range of 400 to 4000 cm^−1^ using a diamond crystal at an angle of incidence of 45°.

#### 2.3.2. Field emission scanning electron microscope

The morphology of the PU–RH composite was analyzed by FE-SEM (Hitachi S-4800, Tokyo, Japan at a voltage of 2.0–15.0 kV. The specimens were sputter-coated with a conductive layer of platinum.

#### 2.3.3. Thermogravimetric Analysis

The TGA was carried out using a Q500 Universal V4.5A (TA instruments, New Castle, DE, USA) by heating from room temperature to 800 °C at a rate of 10 °C/min in air atmosphere.

#### 2.3.4. Flame-Retardant Test

The fire retardancy properties were determined by LOI tests with sample dimensions of 130 × 10 × 10 mm^3^ (Qualitest, ASTM D2863) and UL-94 tests with specimen bars having dimensions of 127 × 13 × 10 mm^3^ (ASTM D3801-96 for vertical burning (UL-94 V) and ASTM D635-98 for horizontal burning (UL-94 HB)). The cone calorimetry (Fire Testing Technology) was carried out according to ISO 5660-1. Samples with dimensions of 100 × 100 × 10 mm^3^ were wrapped in an aluminum foil and exposed to an external heat flux of 50 kW/m^2^.

#### 2.3.5. Mechanical Test

The compression strengths of composite samples with the dimensions 50 × 50 × 25 mm^3^ were measured at a loading rate of 2.5 mm/min using a universal testing machine (AG-X Plus, Shimadzu, Kyoto, Japan) according to ASTM D1621.

#### 2.3.6. Density Measurement 

The apparent density was determined as the ratio between the mass and volume of the PUf composite according to ISO 845:2006. The dimensions of the samples were 50 × 50 × 25 mm^3^.

#### 2.3.7. Measurement of Sorption Isotherms 

The sorption isotherms were acquired by the desiccator method. The adsorption and desorption scanning curves were determined according to the EN ISO 12571 standard. Different saturated salt solutions were used to control the relative humidity. Foam samples with dimensions of 50 × 20 × 10 mm^3^ were dried in an oven at 60 °C for 24 h, their weights were recorded, and they were then set in desiccators with different controlled relative humidities at room temperature. Relative humidities of 33%, 55%, and 75% were employed. When the samples reached the equilibrium states, their weights were again recorded and they were then moved to another desiccator with a different humidity. Using this method, the adsorption and desorption isotherm curves were recorded. Further details of the measurement are presented in the literature [34,35,36].

## 3. Results and discussion

### 3.1. Fire-Retardant Performances

LOI and UL94 tests were carried out to investigate the flammability properties of the PU–RH samples with and without the FRs; the results are presented in Table 2.

**Figure 1:**
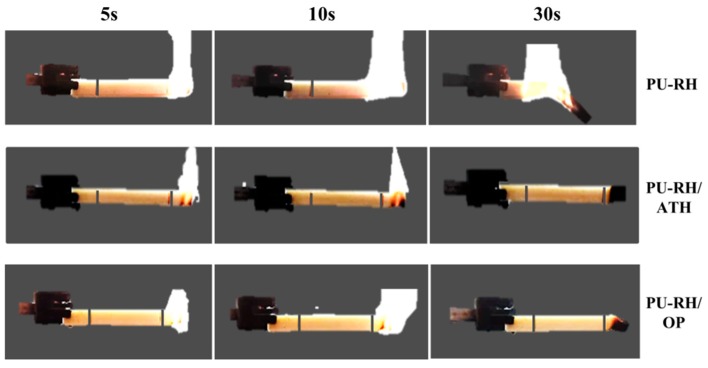
Images of the UL94 HB tests of PU–RH, PU–RH/ATH_125_, and PU–RH/OP_20_.

PU–RH completely burned and failed the UL94 HB test. Both PUf and RH are flammable materials and thus PU–RH could not withstand the fire (Figure 1). In a few seconds, PU–RH extremely burnt over the 1 inch mark and the flame spread to the holding clamp. The 20 php OP or 125 php ATH in PU–RH significantly improved its flame retardancy. The fire ceased completely before reaching the 1 inch mark after 30 s and thus these samples achieved the UL 94 HB rating.

However, the UL94 HB is the lowest standard of the UL94 testing. Therefore, we also carried out a UL94 V test. At the 100 php (27 wt%) ATH loading, the sample achieved the V-1 rating, while the loading reached 125 php (32 wt%), it passed the V-0 rating. Therefore, the minimum content of ATH in PU–RH must be 125 php.

The UL94 V test of both PU–RH samples with 15 php (5 wt%) and 20 php (7 wt%) OP loadings showed that the total burning time was less than 10 s. In addition, dripping was not observed. However, PU–RH/OP_15_ did not pass the UL94 HB test. Therefore, the optimal loading of OP in PU–RH must be 20 php. The samples that passed the UL94 V-0 standard were further evaluated by LOI testing. The minimum oxygen contents for the burning of the PU–RH/ATH_125_ and PU–RH/OP_20_ samples were larger than 21%, whereas the LOI of PU–RH was only 19%.

**Figure 2:**
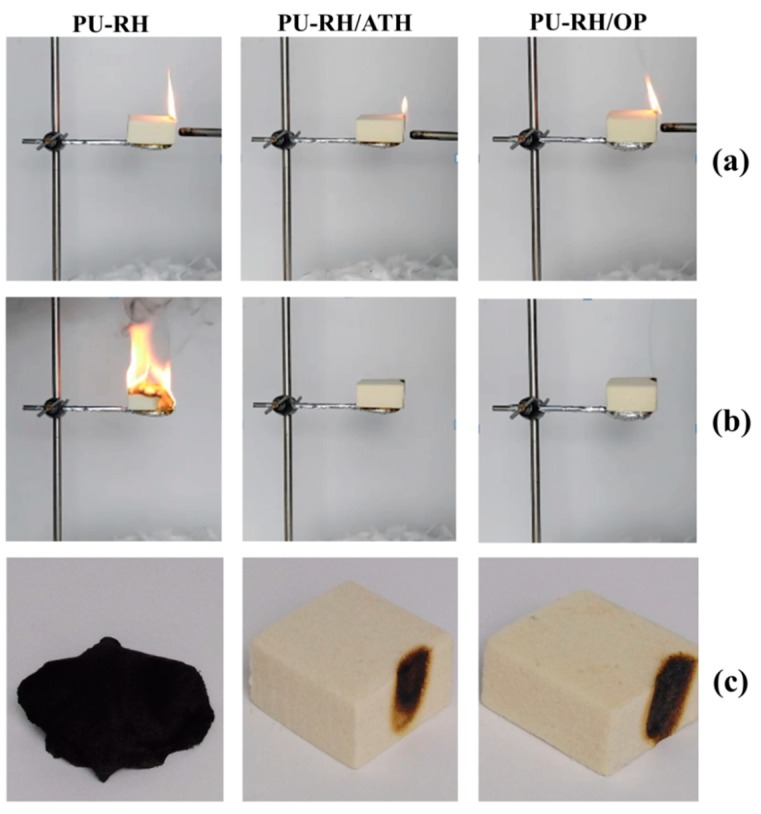
Photographs of the PU–RH and PU–RH/FR mixtures at (**a**) 5 s, (**b**) 10 s, and (**c**) after the combustion.

The rigid PUf is highly combustible, which is its major drawback. Even though the flaming combustion of PU–RH/FRs occurred in less than 10 s, some specimens burned with quite a high flame and it was difficult to clearly observe the FR efficiencies of the samples. Therefore, another test for the foam materials should be carried out. Samples with dimensions of 50 × 50 × 25 mm^3^ were exposed to the fire within 10 s [37]. Figure 2 shows the combustions of the different samples at 5 and 10 s and obtained samples after the burning. The flame was removed at the tenth second. The PU–RH/FRs were self-extinguished, while PU–RH still continued to burn and was finally completely charred. This indicates that the flame retardancy of PU–RH was considerably improved by the loading of ATH or OP.

To obtain the same flame-retardant properties, OP was used in a considerably smaller (7 wt%) quantity than that (32 wt%) of ATH. The mechanism responsible for the effect of OP on the flame retardancy was analyzed in detail [38,39,40,41]. The decomposition of OP formed active radicals in the gas phase, which could capture hydrogen and hydroxide radicals to cease the fire. The condensed phase also contributed to the flame retardancy by generating a phosphorus-containing residual char. ATH acted primarily only in the condensed phase by forming the solid Al_2_O_3_, which protected the inner layer and resisted the oxygen and heat invasion into the matrix. Therefore, OP exhibited a better flame-retardant performance than that of ATH in the PUf composite.

### 3.2. Thermal Decomposition Properties

**Figure 3:**
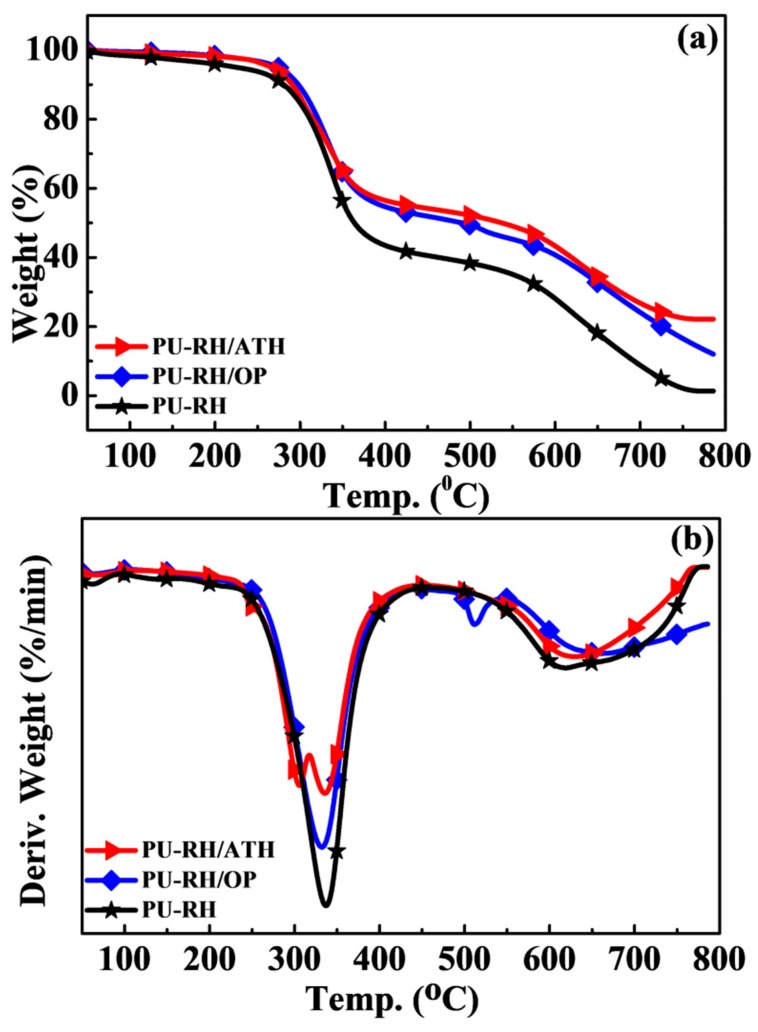
(**a**) TG and (**b**) DTG curves of PU–RH, PU–RH/ATH_125_, and PU–RH/OP_20_ under air.

The thermal stabilities of the PU–RH and PU–RH/FR composites were characterized by TGA in air atmosphere. Figure 3 shows the TG and DTG curves of the PU–RH composites. Table 3 presents TGA data including the 5% mass loss (*T*_5_), 50% mass loss (*T*_50_), and charred residue contents at 550 and 750 °C. The flame retardancy results showed that the optimum FR contents are 20 and 100 php for OP and ATH, respectively. The samples that passed the UL94 V-0 standard were further evaluated and discussed.

Figure 3 shows that both PU–RH and PU–RH/FR mixtures exhibited mainly two-step thermal degradations. Evaporation of volatile molecules, such as water molecules, was observed owing to the high moisture absorptions of the porous PU–RH composites below 250 °C. The first step of the decomposition of PU–RH occurred around 234–412 °C, associated to the breaking of urethane linkages. The second step occurred at 510–760 °C, corresponding to the cleavage of strong bonds such as the aliphatic C–C and aromatic groups, FR, or thermally stable compounds.

Table 3 shows that when the FRs were added, the initial decomposition temperature increased from 234 for PU–RH to 266 and 274 °C for PU–RH/ATH and PU–RH/OP, while the weight losses of the first step decreased from 58% to 43% and 45%, respectively. Particularly, the temperature at the 50% weight loss considerably increased to 537 and 496 °C, respectively, compared to that (364 °C) of PU–RH. The residual char contents of PU–RH, PU–RH/ATH, and PU–RH/OP were 35.0%, 48.7%, and 46.1% at 550 °C. At the higher temperature (750 °C), the TGA curve showed that PU–RH almost completely decomposed leaving a very small amount of residual char (1.3 wt%). The char residue contents of PU–RH/ATH and PU–RH/OP were 22.2 and 17.0 wt%, respectively. These results confirm that the FR increased the thermal stability of the composite by the lower weight loss, shifting it to the higher decomposition temperature and notably higher residual char content than those of PU–RH. As shown in Figure 3b, the DTG curves of PU–RH/ATH and PU–RH/OP indicate that their thermal stabilities are higher than that of PU–RH, as the neat PU–RH thermally degrades with the higher decomposition rate than those of the PU–RH/FRs. This is related to the generation of various thermally stable substances such as aluminum oxide, silicon dioxide, and/or phosphorus-containing compounds during the degradation. The ATH and OP as FRs in PU–RH have large potentials to form a solid char, thus protecting the inner bulk materials.

### 3.3. Fire-Retardant Mode of Action

The flame retardancy of a material strongly depends on the thermal stabilities of the individual components including the FR additives, their interactions, and noncombustible residue formed during the combustion.

**Figure 4:**
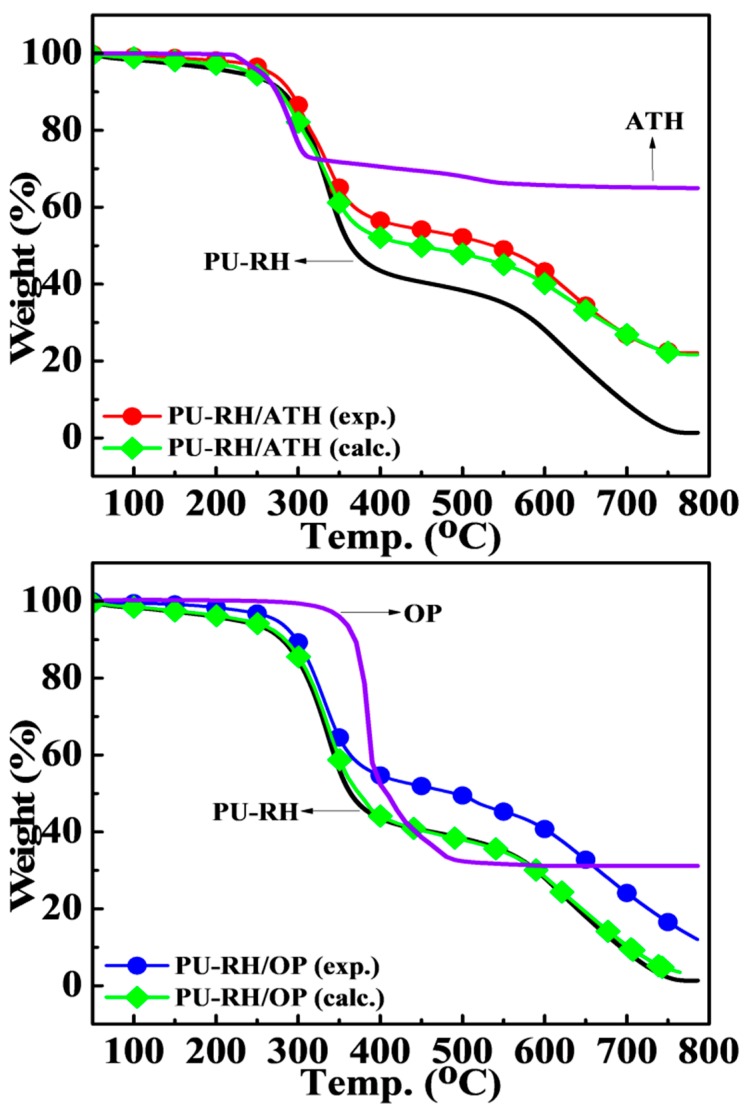
Comparison of the experimentally determined TG curves and values calculated using the additive rule for the PU–RH/FR mixtures under the air atmosphere.

The experimental and calculated TGA curves are overlapped if almost no interaction exists between the FR and PU–RH. As shown in Figure 4, the two curves of PU–RH/ATH did not considerably differ in all temperature ranges, whereas those of PU–RH/OP were different. The experimental TGA curve was shifted to higher temperatures and the residual char quantity was notably higher than the calculated value, particularly in the temperature range above 350 °C.

**Figure 5:**
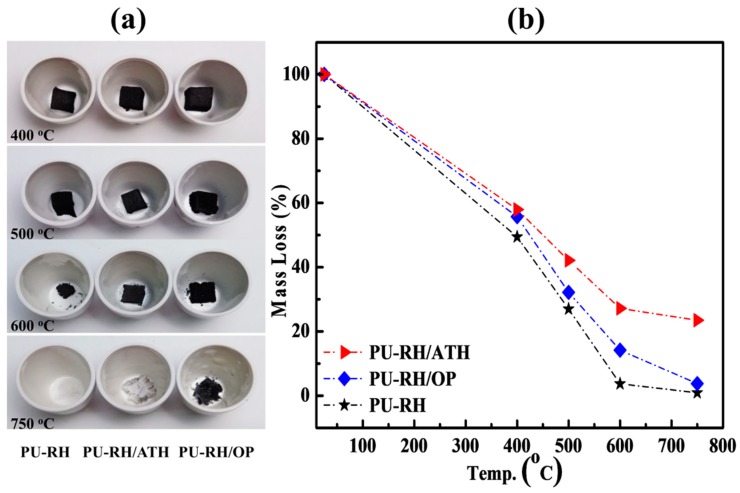
(**a**) Photographs of the char residues of PU–RH, PU–RH/ATH, and PU–RH/OP and (**b**) mass loss curves after the incinerations in the furnace at different temperatures.

To further understand the thermal and flame-retardant properties, the PU–RH and PU–RH/FR samples were incinerated in a furnace at various temperatures; the relevant outcomes are presented in Figure 5. In general, the thermal degradation of each sample occurred mainly between 300 and 750 °C by the fragmentation of weak linkages, such as those at the urethane and/or ester/ether of the polymer chain, or by the decomposition of the FR and products formed by the interaction of PU–RH and FR during the combustion.

A very small amount of white char, silica generated by the degradation of the RH in PU–RH (Figure 5a), remained at 750 °C (0.9%) (Figure 5b). At the same temperature, PU–RH/ATH exhibited a larger white residue content (23.5%). During the thermal degradation, the ATH decomposed endothermically, released water, and produced aluminum oxide as a white powder on the surface. This solid layer acted as a protective layer, preventing the heat and oxygen transfer into PU–RH. In contrast, the OP acted mainly in the gas phase through the release of diethylphosphinic acid, which created active radicals capturing the other fire-formed radicals in the combustion zone. In the condensed phase, the decomposition of the OP generated aluminum phosphate and/or phosphorus-containing char residue. These results are consistent with the above TGA results.

**Figure 6:**
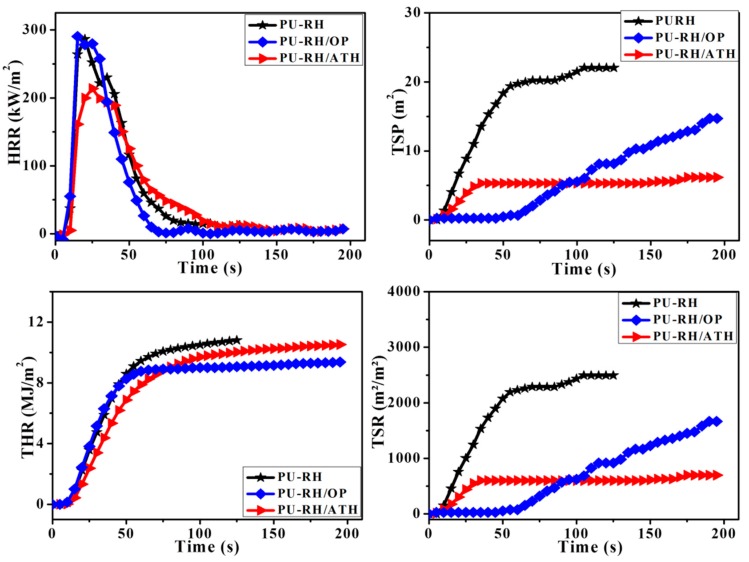
Cone calorimetry results for PU–RH, PU–RH/ATH, and PU–RH/OP.

Cone calorimetry is one of the most effective methods for the evaluation of the fire behaviors of materials, particularly those leaving high residual char contents upon the combustion. The peak heat release rate (PHRR), total heat release (THR), total smoke production (TSP), and total smoke release (TSR) values of the samples are shown in Figure 6. The PHRRs of PU–RH and PU–RH/OP were 287 and 290 kW/m^2^, respectively, while that of PU–RH/ATH was 214 kW/m^2^. The PHRR of PU-RH/ATH_125_ was decreased by 34.1%, compared to that of PU–RH. Notably, the OP did not decrease the PHRR, probably owing to its flame-retardant mechanism. The OP acted in both phases [42,43,44,45,46], but the gas phase was mainly responsible for the mode of action. The sample with the rather low loading of OP (7 wt%) exhibited an excellent fire retardancy. Furthermore, even though the TG curve of PU–RH/OP showed a high residual char content (41%) at 600 °C, the char content was significantly decreased at 750 °C (12%). The char residue content determined by cone calorimetry was 3.8% (Figure S1). These results demonstrate that the OP is expected to act in both gas and condensed phases, but mainly the gas phase is involved in the mode of action. In addition, the char layers may not be sufficiently stable to endure further thermo-oxidative decomposition under the cone calorimetry conditions and consequently do not considerably contribute to the PHRR decrease of PU–RH/OP upon the combustion.

The THR, TSR, and TSP of PU–RH/ATH were lower than those of PU–RH. The lower TSR and TSP correspond to a lower smoke risk. However, after 100 s of combustion, the TSR and TSP of PU–RH/OP were higher than those of PU–RH/ATH. These results are understandable because the ATH mainly acts in the condensed phase and leaves the highest content of a stable residual char above 700 °C (Figure 1a and Figure 5b), while the main gas-phase fire-retardant mechanism of OP involved the release of phosphorus-containing moieties during the combustion, yielding the enhanced smoke release behavior of PU–RH/OP.

The FTIR spectra (Figure S2) of the PU–RH char show the absorption bands of N–H (3564 cm^−1^), O–H (3355 cm^−1^), Si–O (1106 cm^−1^), and Si–OH (832 cm^−1^). The FTIR spectra of the PU–RH/ATH and PU–RH/OP residues show an additional band of Al–O (668 cm^−1^) related to the aluminum oxide/aluminum phosphate in the ATH- and OP-decomposed structures. Moreover, the peaks at 1613 and 1090 cm^−1^ are attributed to the P–OH and P–O stretching bands in PU–RH/OP, respectively. These findings further confirm the FR abilities of the ATH and OP in the formation of the char layers. These layers could function as a barrier inhibiting the thermal and oxygen transport and preventing the inner side of the materials from the direct exposure to the heat and combustion zone.

**Figure 7:**
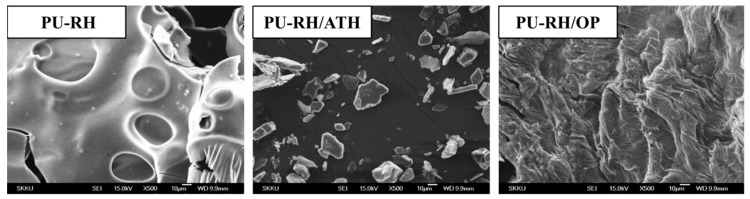
FE-SEM images of the residual chars of PU–RH, PU–RH/ATH_125_, and PU–RH/OP_20_ after the cone calorimetry test.

The morphologies of the charred residues of PU–RH, PU–RH/ATH, and PU–RH/OP are presented in Figure 7. PU–RH exhibited a porous and brittle char layer on the surface with a large number of fractures, while the outer surface residual char of PU–RH/ATH was dense. On the contrary, the OP in PU–RH generated the compact and thick solid char, which contributed to the resistance to the further thermal degradation of the polymeric matrix. These layers prevented the exposure of the interior materials with the attack of oxygen and heat sources to the combustion zone.

Table 4 shows the surface elemental compositions of the residues obtained by the cone calorimetry. The ATH and OP FRs contain aluminum and thus the XPS showed the signal of this element. The peak of phosphorus (13.11%) observed for the PU–RH/OP char indicates that this element existed in the nonvolatile state, and consequently was active in the condensed phase. The higher oxygen element contents in the PU–RH/ATH and PU–RH/OP chars (59.71% and 50.55%, respectively) could be explained by the presence of an inorganic oxide or salt, such as aluminum oxide or aluminum phosphate, whereas the PU–RH char consisted only of a carbonaceous residue with a lower oxygen content (16.71%).

**Figure 8:**
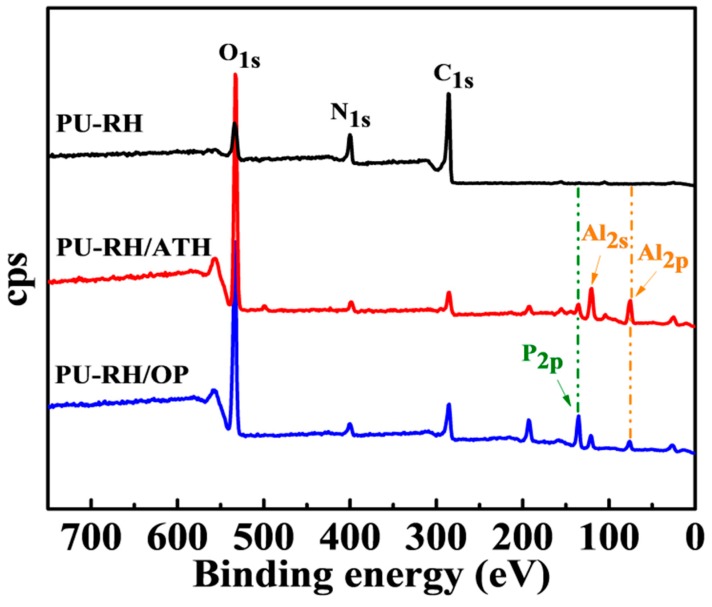
XPS of the PU–RH, PU–RH/ATH, and PU–RH/OP residual chars after the cone calorimetry.

**Figure 9:**
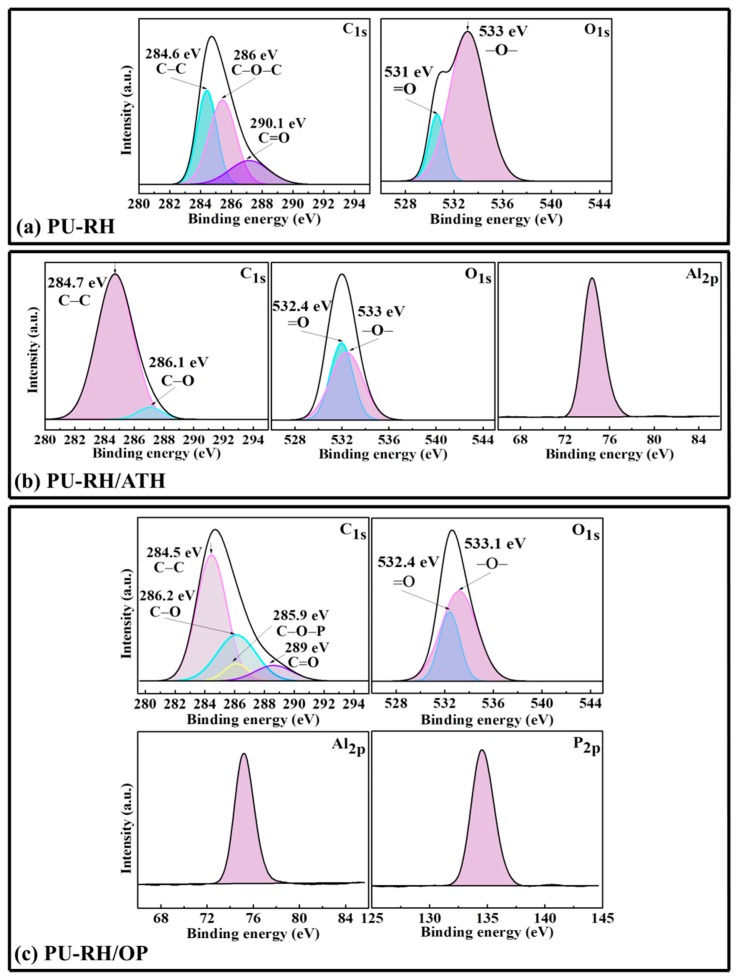
C_1*s*_, O_1*s*_, Al_2*p*_, and P_2*p*_ XPS of the residual chars after the cone calorimetry of (**a**) PU–RH, (**b**) PU–RH/ATH, and (**c**) PU–RH/OP.

Figure 8 shows new peaks at 136.5, 119.8, and 74.6 eV, corresponding to the P_2*p*_-, Al_2*s*_-, and Al_2*p*_-binding energies of the PU–RH/ATH and PU–RH/OP residual chars, respectively. As presented in Figure 9, the C_1*s*_ spectra show peaks at 284.5–284.7, 286.0–286.2, and 289.0–290.1 eV, which could be attributed to C–C and/or C–H in the aliphatic and aromatic species, C–O (ether and/or hydroxyl groups), and C=O group, respectively [47,48]. A distinct C–O–P signal of hydrocarbon phosphate was observed at 285.9 eV in the C_1*s*_ spectrum of PU–RH/OP. The O_1*s*_ spectra contained a peak at 531.0–532.4 eV, which could be attributed to O=C and/or O=P, while the peak at 533.0–533.1 eV is assigned to -O- in the C–O–C, C–O–P, and/or C–OH groups [49,50]. The Al_2*p*_-binding energy of PU–RH/ATH and PU–RH/OP was 74.6 eV, which corresponds to Al_2_O_3_ and/or aluminum phosphate (Figure 9b,c). The peaks between 132.8 and 136.5 eV in the P_2*p*_ spectra could be attributed to the P–O–C and/or PO_3_^−^ groups in pyrophosphate and/or polyphosphate and/or P_2_O_5_ (Figure 9c) [51]. The XPS results demonstrate that PU–RH/ATH produced Al_2_O_3_, which covered the surface of the material, and that PU–RH/OP generated a phosphorus-containing residue during the decomposition, which could act as a heat-resistant char leading to high thermal stability and excellent flame retardancy.

The TGA, FTIR spectroscopy, SEM, XPS, cone calorimetry, and residual char analysis results indicated that the addition of ATH generated a white solid layer (aluminum oxide) scattered over the residue surface. The pyrolysis products of OP, such as the phosphinic/phosphoric acid and/or phosphorus-containing moieties, would self-condense or react with other volatiles generated by the decomposition of PU–RH to form the stable and continuous phosphorus/aluminum-rich residual chars, which exhibit barrier effects inhibiting the transport of heat and oxygen.

### 3.4. Physical and Mechanical Properties

**Figure 10:**
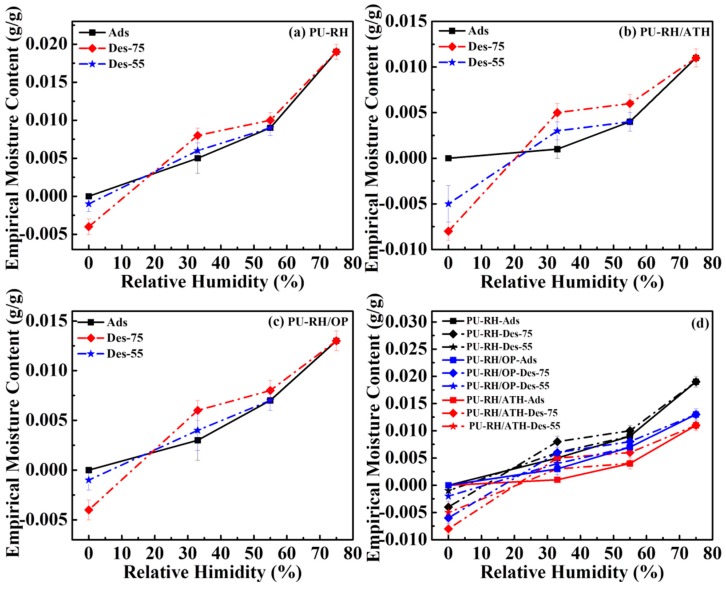
Sorption isotherms of (**a**) PU–RH, (**b**) PU–RH/ATH, (**c**) PU–RH/OP, and (**d**) all samples.

The inclusion of FRs into foam materials strongly influences their physical and mechanical properties as well as practical applications. Therefore, the apparent density, compressive strength, and moisture sorption were investigated; the relevant results are shown in Table 5 and Figure 10.

ATH and OP are high-density additives, and thus their presence in the polymer matrix led to the increase in PU–RH apparent density from 57 to 85 and 59 kg/m^3^ for PU–RH/ATH and PU–RH/OP, respectively.

Nevertheless, the addition of the FR led to the decrease in compressive strength of the foam. The addition of the ATH to PU–RH led to a significant increase in density. The ATH largely contributed considering its high content (up to 32 wt%) and high density (2.42 g/cm^3^). Assuming the ATH did not change the foam formation and the density of PU–RH, the theoretical density of the composite must be 85 kg/m^3^. This value is almost equal to the experimental value of 84.94 kg/m^3^. Therefore, the addition of ATH did not affect the foam formation of PU or the density of the PU–RH foam did not change. The reduction in compressive strength of PU–RH/ATH_125_ compared to that of PU–RH could be attributed to the irregularity of the pore sizes of PU–RH/ATH_125_, which could only withstand the lower strength. The high loading of ATH, a high-density inorganic compound, could not easily evenly disperse in polyol and PU–RH, which interfered with the normal foam formation. Figure S3 shows the large pore size distribution of PU–RH/ATH_125_. Upon the addition of OP, the larger pore sizes of PU–RH/OP also decreased its yield strength compared to that of PU–RH.

The adsorptions and desorptions of the neat PU–RH and PU–RH/FR mixtures were studied by the desiccator method. All samples were investigated at different relative humidities of 33%, 55%, and 75%. The equilibrium moisture content (mass (g/g)) of each sample was calculated as [34,35,36]

(1) $$u=(m-m_{o})/m_{o},$$

where *m* is the mass of the specimen at the moisture stage and *m*_o_ is the mass at the oven-dried stage. The relevant main adsorption and desorption curves are presented in Figure 10.

For most materials, the desorption to equilibrium leads to a higher moisture content than that upon the adsorption to equilibrium under the same ambient climate conditions [52]. PU–RH exhibited a higher moisture absorption ability than those of PU–RH/OP and PU–RH/ATH. As additives filled in the polymeric matrix, the FRs interrupted the exposure of the foams to the moisture environment and led to the decreased adsorption ability.

In the humidity range of 20%–75%, all isothermal sorption curves exhibited hysteresis, in the order of PU–RH/ATH > PU–RH/OP > PU–RH, owing to the reduction of interaction between the moisture and FRs. At a humidity below 20%, particularly at 0%, the empirical moisture content of the desorption curve was lower than that of the adsorption curve. This unusual behavior can be explained by the effect of the RH in the composite. The chemical composition of the RH included approximately 20% silica, 30% lignin, and 40% cellulose [53,54], in which the organic components were often degraded by microorganisms under the high humidity. In this study, the samples were exposed to a highly humid atmosphere for a long time (about six weeks) and thus the organic components of the composites were decomposed into volatile materials, such as CO_2_ and H_2_O, reducing the sample weights compared to those of the original samples. Particularly, the FR enhanced this bio-decomposition (0.4% of PU–RH, 0.5% of PU–RH/OP, and 0.7% of PU–RH/ATH) because it could create an acidic medium, which accelerated the biodegradation.

## 4. Conclusions

The composites of PUf reinforced with RH, an agricultural waste product, and nonhalogen FRs are of significance for the improvement of fire safety and smoke suppression. In this study, the loading of a rather low OP content (7 wt%) significantly improved the flame retardancy and thermal stability of PU–RH. The minimum ATH loading content in PU–RH must be 32 wt%. All PU–RH/FR samples achieved the UL94 V-0 rating. Their LOI values reached 22%–23%. Their PHRR, THR, TSP, and TSR values were decreased. The cone calorimetry results were in good agreement with the UL94 and TGA results. The compressive strengths of PU–RH/FRs were decreased compared to that of PU–RH. The sorption isotherms of PU–RH and PU–RH/FRs exhibited hysteresis loops in the humidity range of 20%–75%. PU–RH/FRs had lower moisture sorption abilities than that of PU–RH.

The formulation with ATH created a considerable residual char content, inhibiting the transport of heat, oxygen, and fuel. In contrast, the OP mainly acted in the gas phase by releasing active radicals, which were able to capture the other radicals in the combustion zone. In the condensed phase, the decomposition of the OP generated phosphorus/aluminum-rich residual chars. The remarkable improvements in flame retardancy and thermal properties of PU–RH could pave the way for an environmentally friendly development and overcoming the major disadvantage of the highly flammable PUf.

## Figures and Tables

**Table 1:** Main formulations by wt/wt and php of rice husk-reinforced polyurethane (PU–RH) samples with and without flame retardants (FR).

Samples	PU–RH*/FR (wt/wt)	ATH (php)	OP (php)
PU–RH	100.0/0.0	-	-
PU–RH/ATH_100_	73.0/27.0	100	-
PU–RH/ATH_125_	68.0/32.0	125	-
PU–RH/OP_15_	95.0/5.0	-	15
PU–RH/OP_20_	93.0/7.0	-	20

* Polyol/MDI: 1.0/1.5 (g/g), RH: 5 wt%; water: 2 php (parts per hundred of polyol by weight).

**Table 2:** UL-94 and LOI results for the PU–RH and PU–RH/FR mixtures.

Samples	PU–RH/FR(wt/wt)	LOI(%)	UL94 HB	UL94 V		
				t_1_/t_2_^a^	Dripping	Rating
PU–RH	100.0/0.0	19	Fail	No rating		
PU–RH/ATH_100_	73.0/27.0	-	HB	8/7	No	V-1
PU–RH/ATH_125_	68.0/32.0	23	HB	1/1	No	V-0
PU–RH/OP_15_	95.0/5.0	-	77 mm/min (Fail)	5/1	No	*
PU–RH/OP_20_	93.0/7.0	22	HB	2/1	No	V-0

a: t_1_ and t_2_ (s): the average time after removing the flame at the 1st ignition and 2nd ignition* Samples burnt with the flame up to the specimen holding clamp.

**Table 3:** Thermal stability parameters obtained by the TGA.

Samples	PU–RH/FR (wt/wt)	T_5_ (°C)	T_50_ (°C)	Residue at 550 °C (%)	Residue at 750 °C (%)
PU–RH	100/0	234	364	35.0	1.3
PU–RH/ATH_125_	68/32	266	537	48.7	22.2
PU–RH/OP_20_	93/7	274	496	46.1	17.0

**Table 4:** Surface elemental compositions of the PU–RH samples.

Samples	C*_1s_* (%)	O*_1s_* (%)	Al*_2p_* (%)	P*_2p_* (%)
PU–RH	81.66	16.71	-	-
PU–RH/ATH_125_	15.73	59.71	25.1	-
PU–RH/OP_20_	26.42	50.55	9.93	13.11

**Table 5:** Physical and mechanical properties of the PU–RH and PU–RH/FR mixtures.

Samples	PU–RH/FR (wt/wt)	Apparent Density (kg/m^3^)	Compressive Strength (kPa)
PU–RH	100/0	57.02 ± 0.53	219.96 ± 11.80
PU–RH/ATH_125_	68/32	84.94 ± 0.67	164.96 ± 17.42
PU–RH/OP_20_	93/7	58.62 ± 1.07	152.91 ± 4.80

## Supplementary Materials

The following are available online at https://www.mdpi.com/2073-4360/11/10/1587/s1, Figure S1: The digital photos of the residual char after cone calorimetry test of (a) PU–RH, (b) PU–RH/ATH, and (c) PU–RH/OP; Figure S2: FTIR spectra of PU–RH and PU–RH/FR residues after cone calorimetry test; Figure S3: SEM micrographs of PU–RH and PU–RH/FR residues after cone calorimetry test.
