# Supplementary material for: Excellent Fireproof Characteristics and High Thermal Stability of Rice Husk-Filled Polyurethane with Halogen-Free Flame Retardant
Source: Polymers (Basel). 2019 Sep 28;11(10):1587. doi: 10.3390/polym11101587 (PMC6835888; doi:10.3390/polym11101587)
Supplement: Supplementary file 1 [file polymers-11-01587-s001.pdf]

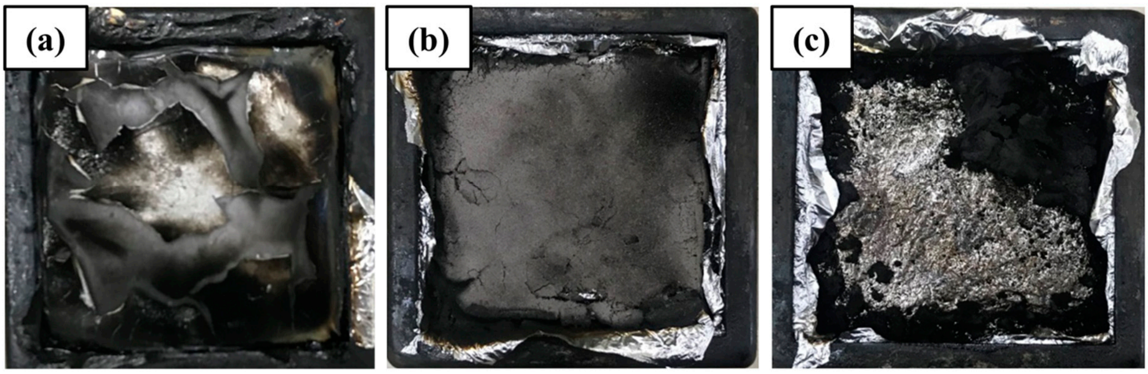

Figure S1. The digital photos of the residual char after cone calorimetry test of (a) PU–RH, (b) PU–RH/ATH, and (c) PU–RH/OP.

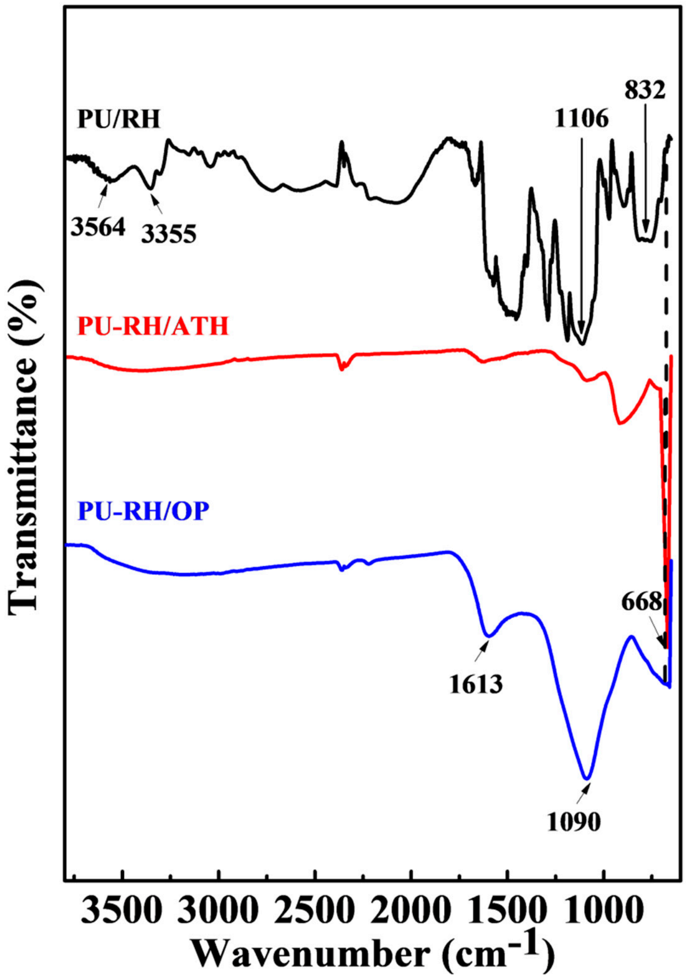

Figure S2. FTIR spectra of PU–RH and PU–RH/FR residues after cone calorimetry test.

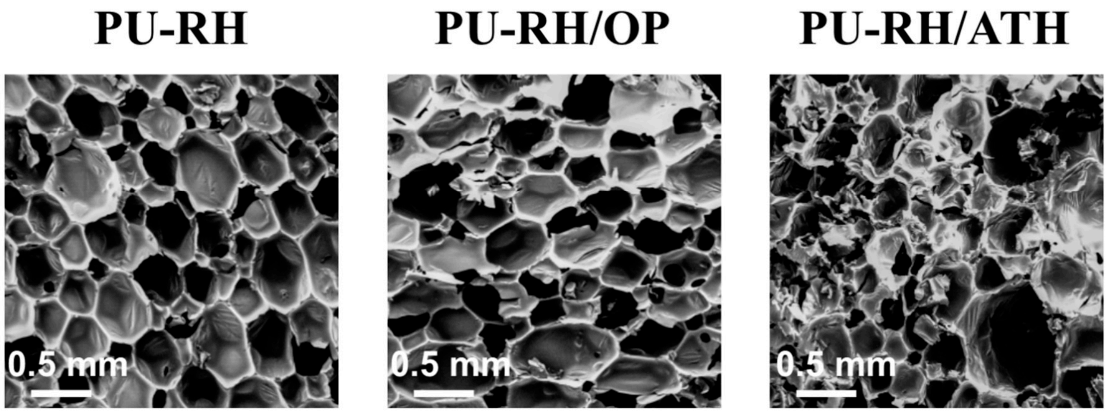

Figure S3. SEM micrographs of PU–RH and PU–RH/FR residues after cone calorimetry test.
